# Supplementary material for: Independent duplications of the Golgi phosphoprotein 3 oncogene in birds
Source: Sci Rep. 2021 Jun 14;11:12483. doi: 10.1038/s41598-021-91909-6 (PMC8203631; doi:10.1038/s41598-021-91909-6)
Supplement: Supplementary file 1 — Supplementary Information. [file 41598_2021_91909_MOESM1_ESM.zip › Supplementary_Information/Supplementary_Fig_1.pdf]

## GOLPH3

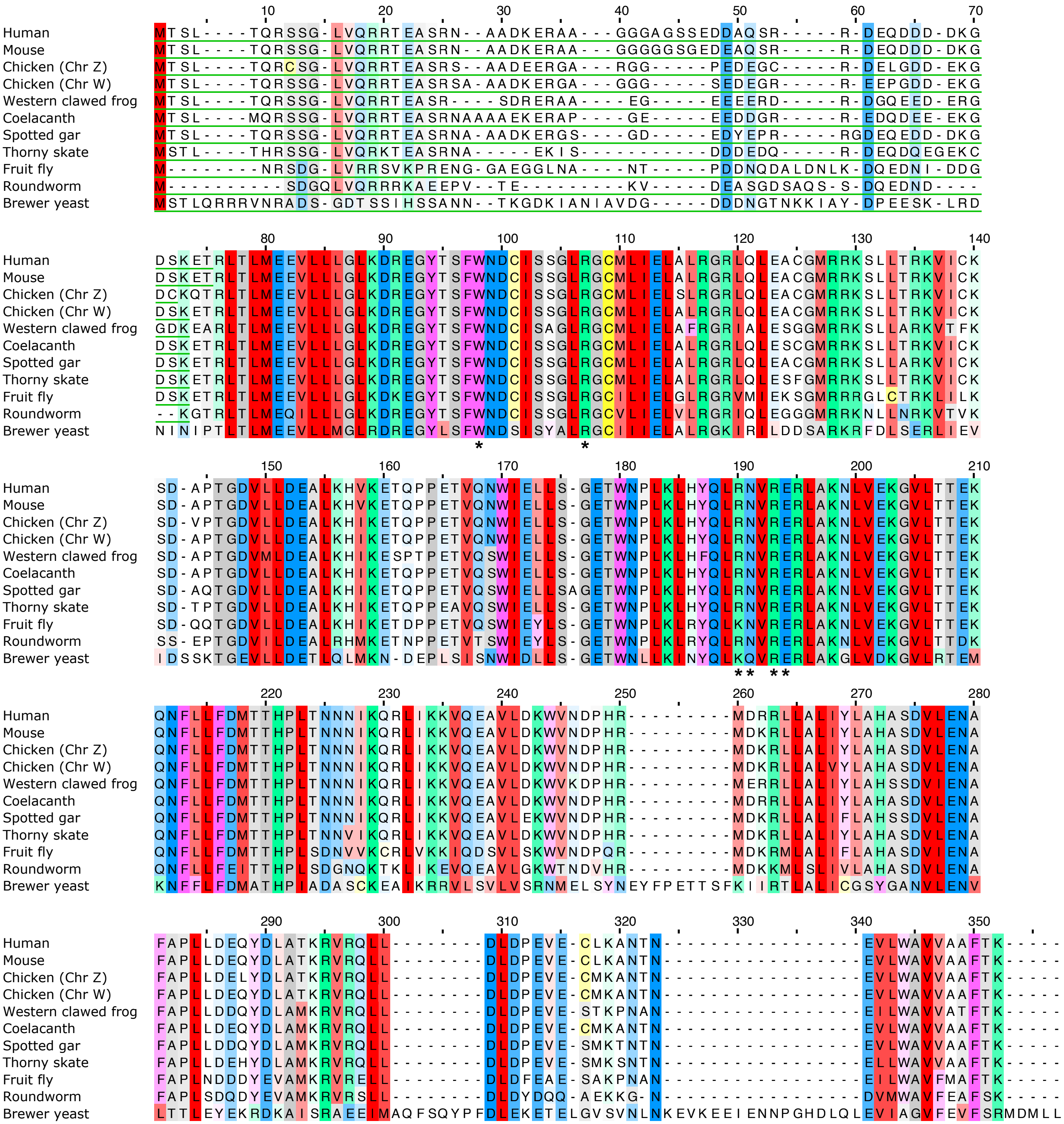


**Supplementary Figure 1.** Multiple sequence alignment of GOLPH3 from human (*Homo sapiens*), mouse (*Mus musculus*), chicken (*Gallus gallus*), Western clawed frog (*Xenopus tropicalis*), coelacanth (*Latimeria chalumnae*), spotted gar (*Lepisosteus oculatus*), thorny skate (*Amblyraja radiata*), fruit fly (*Drosophila melanogaster*), roundworm (*Caenorhabditis elegans*) and brewer yeast (*Saccharomyces cerevisiae*). In chicken, chromosome Z (Chr Z) encodes GOLPH3.1$_{GA}$ and chromosome W (Chr W) encodes GOLPH3.2$_{GA}$. Underlined in green are residues predicted to be part of the N-terminal disordered regions as shown in Figure 6A. Asterisks highlight conserved residues involved in GOLPH3 binding to phosphatidylinositol 4-phosphate. Color scheme and colors with different levels of saturation, representing different levels of amino acid conservation, are defined as implemented in Jalview software: A, G, P, S, T in gray; H, K, R in green; D, E, N, Q in blue; C in yellow; F, W, Y in magenta.
