# Supplementary material for: Independent duplications of the Golgi phosphoprotein 3 oncogene in birds
Source: Sci Rep. 2021 Jun 14;11:12483. doi: 10.1038/s41598-021-91909-6 (PMC8203631; doi:10.1038/s41598-021-91909-6)
Supplement: Supplementary file 1 — Supplementary Information. [file 41598_2021_91909_MOESM1_ESM.zip › Supplementary_Information/Supplementary_Fig_2.pdf]

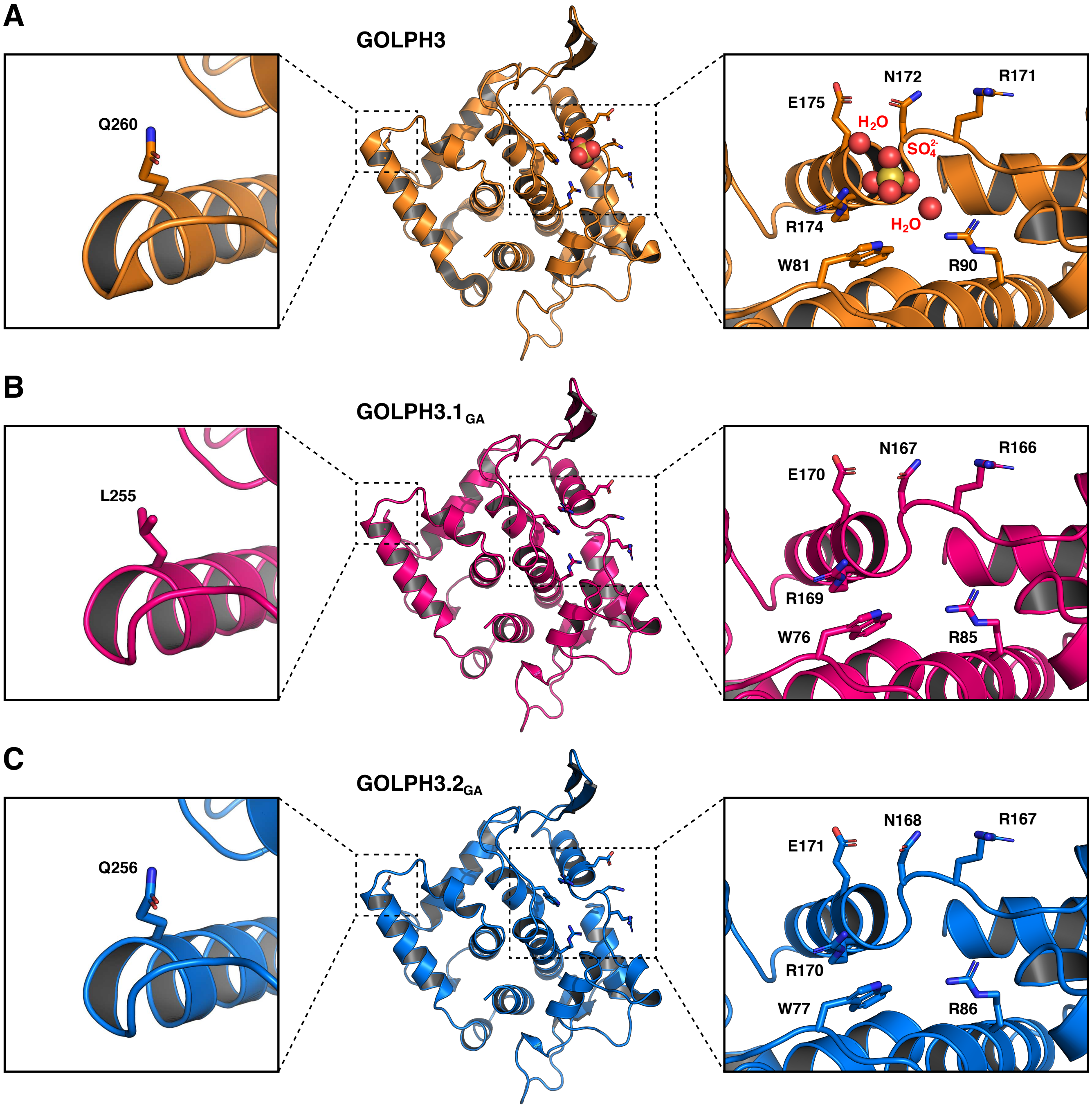

Supplementary Figure 2. Ribbon representation of the crystal structure of N-terminal truncated, human GOLPH3 (A; PDB entry 3KN1) and of homology models of GOLPH3.1$_{GA}$ (B) and GOLPH3.2$_{GA}$ (C) from chicken (*Gallus gallus*). The insets highlight non-conservative divergent L255 in GOLPH3.1$_{GA}$ compared to human GOLPH3 and GOLPH3.2$_{GA}$ (left in A-C) and conservation of residues involved in binding to phosphatidylinositol 4-phosphate (right in A-C).
