# Supplementary material for: Independent duplications of the Golgi phosphoprotein 3 oncogene in birds
Source: Sci Rep. 2021 Jun 14;11:12483. doi: 10.1038/s41598-021-91909-6 (PMC8203631; doi:10.1038/s41598-021-91909-6)
Supplement: Supplementary file 1 — Supplementary Information. [file 41598_2021_91909_MOESM1_ESM.zip › Supplementary_Information/Supplementary_Fig_3.pdf]

GOLPH3L

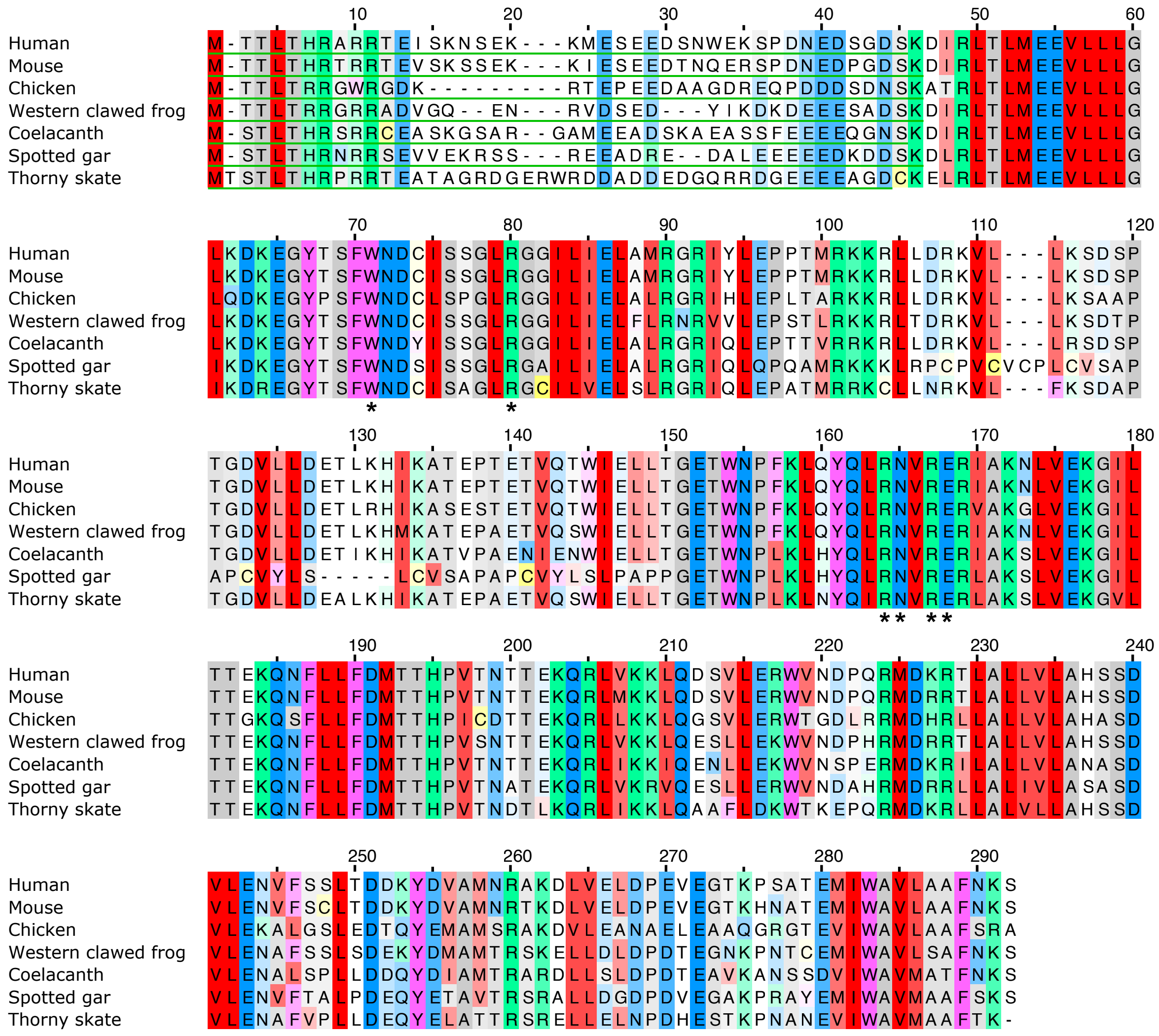

Supplementary Figure 3. Multiple sequence alignment of GOLPH3L from human (*Homo sapiens*), mouse (*Mus musculus*), chicken (*Gallus gallus*), Western clawed frog (*Xenopus tropicalis*), coelacanth (*Latimeria chalumnae*), spotted gar (*Lepisosteus oculatus*) and thorny skate (*Amblyraja radiata*). Underlined in green are the residues predicted to be part of the N-terminal disordered regions as shown in Figure 6B. Asterisks highlight conserved residues involved in GOLPH3 binding to phosphatidylinositol 4-phosphate. Color scheme and colors with different levels of saturation, representing different levels of amino acid conservation, are defined as implemented in Jalview software: A, G, P, S, T in gray; H, K, R in green; D, E, N, Q in blue; C in yellow; F, W, Y in magenta.
